# Cell-mediated and humoral immune responses in pigs following primary and challenge-exposure to *Lawsonia intracellularis*

**DOI:** 10.1186/1297-9716-43-9

**Published:** 2012-02-08

**Authors:** Henriette Cordes, Ulla Riber, Tim K Jensen, Gregers Jungersen

**Affiliations:** 1 National Veterinary Institute, Technical University of Denmark, Bülowsvej 27, 1790 Copenhagen V, Denmark

## Abstract

To investigate immune responses upon re-infection with *Lawsonia intracellularis*, local and peripheral humoral and cell-mediated immune responses to primary and challenge inoculations were studied in 22 pigs. Pigs were orally inoculated with virulent *L. intracellularis *at the age of 5-6 weeks, treated with antibiotics and challenged with a re-inoculation (RE) at the age of 12 weeks. Treatment control (TC) pigs received only the primary inoculation and challenge control (CC) pigs received only the secondary inoculation at 12 weeks of age. Following this regimen, all RE pigs were protected against the re-infection as defined by reduced colonisation and pathology of intestinal mucosa, absence of bacterial shedding and without increase in serum acute phase protein response. In the protected RE pigs, serum IgG responses were variable with both high and low responders. Serum IgA responses were not boosted by the re-inoculation, since identical intestinal IgA responses developed in response to the inoculation in both the susceptible CC pigs and the protected RE pigs. A memory recall cell-mediated immune response developed in RE pigs which was significantly stronger compared to the primary response in age-matched CC pigs as assessed by whole blood IFN-γ assay and by calculation of IFN-γ integrated median fluorescence intensity (iMFI) after flow cytometry. The major IFN-γ producing cells were identified as CD8^+ ^and CD4^+^CD8^+ ^double positive lymphocytes. The results indicate that cell-mediated immune responses are likely mediators of protective immunity against *L. intracellularis*, with CD8^+ ^effector cells and CD4^+^CD8^+ ^double positive memory T cells as main contributors to the antigen-specific IFN-γ production.

## Introduction

*Lawsonia intracellularis *is an obligate intracellular bacterium residing in intestinal epithelial cells and identified as the aetiological agent of porcine proliferative enteropathy [1-3]. The manifestations of proliferative enteropathy are one of the most important diseases of modern pig production with decreased animal welfare, increased antibiotic usage and substantial economic losses. It has previously been shown that *L. intracellularis *infection and disease are reduced after vaccination [4] or re-infection [5,6], but the immunologic responses to infection with *L. intracellularis *have not been fully elucidated.

In pig herds infected with *L. intracellularis*, seroconversion is generally observed between 9-24 weeks of age or approximately 1-2 weeks after detection of faecal shedding of *L. intracellularis *[7,8]. In experimental infection studies, seroconversion is also generally measured from 1-2 weeks after the beginning of faecal shedding. The antibody response persists for a limited time-period (2-3 months) and consists mainly of early IgM antibodies, indicating active disease, followed by development of IgG antibodies [9-11]. *L. intracellularis*-specific IgA has been reported to be limited in serum from infected pigs, but by immunocytochemistry local non-specific IgA has been detected close to *L. intracellularis *within enterocytes [12,13]. Guedes and Gebhart [14] have shown that *L. intracellularis*-specific mucosal IgA can be detected in intestinal lavage 3 weeks post *L. intracellularis *inoculation of pigs.

Although local inflammatory responses to *L. intracellularis *infection are minimal, cell-mediated immune responses (CMI) with infiltration of CD8^+ ^cells and macrophages have been observed in intestinal sections from pigs affected with proliferative enteropathy [13]. More recently, however, MacIntyre et al. [15] demonstrated an association between the presence of *L. intracellularis *and reduced numbers of T and B cells in intestinal tissue sections, suggesting an immunosuppressive mechanism of *L. intracellularis *infection. Given the intracellular nature of *L. intracellularis*, CMI are expected to be significant and it has been clearly shown that IFN-γ produced by T cells plays an important role in limiting *L. intracellularis *pathology in a mouse model [16]. However, quantification of *L. intracellularis*-specific CMI in pigs has proven to be difficult, since both delayed type hypersensitivity (DTH) skin tests and in vitro IFN-γ responses appear to be of low level and only measurable in a subset of infected pigs [14,17]. Recently, however, we reported how potentiating the whole-blood IFN-γ assay with exogenous recombinant IL-18 enhanced the antigen-specific production of IFN-γ sufficiently to facilitate a more detailed measurement of CMI development in *L. intracellularis *infected pigs [18].

We have previously demonstrated protection against re-infection in primary infected pigs compared to age matched challenge control pigs as evidenced by reduced colonisation and pathology of intestinal mucosa, absence of bacterial shedding and without increase in acute phase protein response [6]. Here we report detailed studies on humoral and CMI responses after both primary and secondary *L. intracellularis *infections in these pigs.

## Materials and methods

All chemicals were purchased from Sigma-Aldrich, Denmark, unless otherwise indicated.

### Experimental infection of pigs with *L. intracellularis *and sample collection

The experimental design has been described in detail in the previously published re-infection study, Experiment II [6]. Briefly, 22 piglets (age 5-6 weeks at day 0) were randomly assigned to three groups: the Re-infection (RE, *N *= 10) and Treatment control (TC, *N *= 5) groups received an oral primary *L. intracellularis *inoculation at day 0, while pigs in the Challenge control (CC, *N *= 7) group remained naive. All pigs received 7 days of oral tiamulin treatment (7 mg/kg bodyweight, Tiamutin^®^, Novartis, Copenhagen, Denmark) from day 21. At day 49, pigs in the RE and CC groups received an oral *L. intracellularis *challenge inoculation. Doses were approximately 2 × 10^9 ^and 10^10 ^*L. intracellularis *bacteria per pig for the primary and challenge inoculations, respectively. Faeces and blood samples were collected at the time-points indicated and the pigs were sacrificed at day 83 or 84. In a preceding Experiment I, similar groups of pigs in an almost identical study design [6] were used to isolate cells from intestinal tissues at necropsy on day 70 or 74 post primary inoculation (i.e. 3 weeks post challenge), but other immunological data of these pigs are not reported here.

All serum samples were kept below -20°C until further use. Faecal samples were freeze-dried and kept below -20°C for measurement of IgA (see below).

All procedures of animal handling and experimentation were approved by the Danish Animal Experiments Inspectorate.

### Preparation of faeces extracts for IgA measurement

Extracts were made from faeces as described by Haneberg et al. [19] with some modifications. Briefly, 200 mg faeces was lyophilised and dissolved in 10 μL extraction buffer (PBS with 0.05% Tween 20 (PBS-T) with 0.2 mg/mL soybean trypsin inhibitor, 1 mg/mL EDTA and 1 mM phenylmethanesulfonyl fluoride per mg dry faeces and vortexed). The extract was centrifuged at 16 000 × *g *for 10 min. at 21°C, and 10 μL 10% bovine serum albumin in PBS and 10 μL 1% sodium azide solutions were added per mL supernatant before storage below -20°C. Ileum contents collected at autopsy day 83/84 were treated in the same manner for detection of IgA.

### Detection of *L. intracellularis*-specific IgG and IgA by ELISA

Measurements of *L. intracellularis*-specific IgG were done by indirect ELISA using Polysorp plates (Nunc, Roskilde, Denmark) coated with *L. intracellularis *deoxycholate (DOC) extract as previously described [20]. For detection of specific IgA in serum or faeces extracts, Polysorp plates were coated with *L. intracellularis *DOC extract diluted 1:400 and with serum samples diluted 1:12.5 in blocking buffer or with undiluted faeces extracts. The detection step employed incubation with HRP-conjugated anti-porcine IgA (A100-102P, Bethyl, Montgomery, Alabama, USA) diluted 1:10 000 in blocking buffer. IgG and IgA readings are given as OD%, which are OD values normalised by expressing them as % of in-plate values of positive control sera with high content of *L. intracellularis*-specific IgG or IgA.

For specific faecal IgA, the values were expressed as OD% per amount of total IgA in faeces. This was done to correct for variations in homogeneity and water content (due to e.g. diarrhoea). Total IgA in faeces was measured as described for specific IgA with the exception that Maxisorp plates (NUNC) were coated with anti-porcine IgA monoclonal antibodies (AAI40, Serotec, Oxford, UK) diluted 1:6000, faeces extracts were diluted from 1:100 to 1:10 000 in blocking buffer and detection of bound IgA was done with HRP-anti-porcine IgA (AAI40P, Serotec) diluted 1:10 000 in dilution buffer. Concentration of total IgA in faeces extract was calculated by including a pig reference serum with known IgA concentration i.e. 650 μg/mL (RS10-107, Bethyl).

### Measurement of antigen-specific IFN-γ response

As a measure of cell-mediated immune response to *L. intracellularis*, a whole-blood IFN-γ assay potentiated with IL-18 was used [18]. Briefly, samples of 1 mL fresh heparin-stabilised blood were cultured in 24-well-plates (Greiner-Bio One GmbH, Frickenhausen, Germany) for 20-22 h at 37°C in 5% CO_2 _with SDS-treated *L. intracellularis *antigen (SDS-antigen, 5 μg/mL) in the presence of recombinant porcine IL-18 (R&D Systems Europe Ltd., Abingdon, United Kingdom) at 50 ng/mL. IL-18 potentiated cultures with PBS or Staphylococcus enterotoxin B (SEB) at 1 μg/mL were performed in parallel as negative and positive controls, respectively. After culture, the plasma supernatants were collected and stored below -20°C until quantification of released IFN-γ by a monoclonal sandwich ELISA [18]. The antigen-specific IFN-γ response to *L. intracellularis *was calculated as the IFN-γ measured in SDS-antigen cultures subtracted from the IFN-γ measured in the PBS culture for the respective sample. Based on a frequency distribution of IFN-γ measurements in non-inoculated pigs a limit of unspecific response was set to 100 pg/mL.

IFN-γ response in blood samples was measured at the time points indicated (see Figure 3). A few samples were excluded as invalid due to a level of non-specific IFN-γ above 200 pg/mL in PBS cultures.

### Phenotyping of IFN-γ producing cells

IFN-γ producing cells were examined day 48, 67, 75, and 82 by intracellular staining and flow cytometry after culture of peripheral blood mononuclear cells (PBMC) with Ag, SEB or PBS and IL-18 (50 ng/mL) for 20-22 h at 37°C in 5% CO_2_. Cells were stained for CD4 (clone 74-12-4), CD8 (clone 76-2-11, recognising CD8α), CD25 (clone k231.3B2) and IFN-γ (clone cc302) and analysed by 4-colour flow cytometry on FACSCanto II with Diva 6 software (BD Biosciences) with identification of cell populations by Boolean gating. The analysis and processing of data are described in [18]. The cell staining was focused on identifying CD4^+^CD8^+ ^double positive cells, but since CD3 and CD8β staining was not included, the CD8α staining alone cannot discriminate between cytotoxic T lymphocytes (CTLs), TCR-γδ T cells and NK cells which all may express CD8α [21]. Approximately 50 000 lymphocytes were analysed in each sample.

Integrated median fluorescence intensity (iMFI) was calculated as the product of the frequency of IFN-γ producing cells and the median fluorescence intensity of the IFN-γ signal [22]. *L. intracellularis*-specific IFN-γ iMFI was calculated as iMFI measured in Ag-cultures with subtraction of the iMFI in PBS-cultures. A cut-off of 15 was established from the *L. intracellularis*-specific iMFI of CC pigs at day 48 (mean + 2SD). To identify the phenotypes and distribution of IFN-γ producing cells, the frequencies of single CD4^+^(CD8^-^), single CD8^+^(CD4^-^), and CD4^+ ^CD8^+ ^double positive cells, respectively, were identified within the IFN-γ producing population.

The ratio of CD4^+^:CD8^+ ^cells was calculated from the means of frequencies in cultures with Ag and PBS, which were of similar levels after the short 22 h culture.

### Characterisation of intestinal cell subsets

In a preceding similar experiment (Experiment I [6]) intraepithelial (IEC) and lamina propria (LPC) cells were isolated from the intestine of pigs at experimental day 70 or 74. Approximately 15 cm of ileum were tied at one end, inverted, filled with HBSS and tied at the other end. Thereafter the inverted intestines were incubated in Ca/Mg-free HBSS with EDTA at 37°C for 2 h to remove epithelial cells, as described by Bailey et al. [23]. Briefly, IEC were isolated from the interphase after centrifugation of the cell suspension on Percoll (35%-75%). To isolate LPC, the inverted intestines were cut open and mucus was rinsed off with a blunt glass triangle. Then the denuded mucosa was scraped off the muscular layers with a scalpel avoiding Peyer patches. The mucosal scrapings were incubated for 80 min in RPMI 1640 with 100 IU/mL collagenase (Sigma C9263), and the released cells were separated on Percoll (35%-75%).

IEC and LPC were resuspended in flow cytometry wash buffer (PBS containing 0.1% azide and 0.5% BSA) and stained using mAbs against CD3 (clone PPT3), CD4 (clone 74-12-4), CD8 (clone 76-2-11), CD25 (clone K231.3B2), CD172a (clone 74-22-15), and secondary antibodies conjugated with FITC, PE, or PerCP. The stained cells were analysed by 3-colour flow cytometry on FACScan using CellQuestPro software (BD Biosciences).

### Scoring of immune response levels for individual pigs

To obtain unbiased estimates of the level of different immunological responses for statistical analyses, the levels of *L. intracellularis*-specific IgA in faeces and ileum content, IgA and IgG in serum, and IFN-γ-production in whole blood cultures were scored for individual pigs. The parameters for each pig at all time-points (IgA in ileum content only at days 83 or 84) were categorised in three categories: 0 for no response (below cut-off), 1 for low to medium response (above cut-off and below median values) and 2 for high response (above median values). Median values for all parameters were calculated from all positive samples from day 60 to day 82 (except for faeces IgA which was from day 63). The cut-off to median values used were the following: 30-89 OD% for SIgA, 25-62 OD% for serum IgA, 16-54 OD% for serum IgG and 100-1037 pg/mL for IFN-γ. The individual response levels at each sampling (except IgA in ileum content) were summed up for the primary infection period: from day 6 or 11 to day 48, and for the secondary infection period (after challenge inoculation day 49): day 67-75 for humoral immunity or day 60-82 for CMI. These sums of scores were then categorised in response levels: no response, low-medium response and high response for the two time periods. High and low-medium responses were defined as summed scores above or equal/below half of the possible maximum summed score, respectively. Response levels are shown in Table 1. Due to the different numbers of data-points in the different time-periods, it was possible to compare response levels between individual pigs and between groups in the same time-periods only, not between different time-periods.

**Table 1:** Scores of host immune response levels in age-matched pigs with primary and secondary immune responses.

		Humoral immunity							Cell-mediated immunity	
		**Faeces SIgA**		**Ileum SIgA**	**Serum IgA**		**Serum IgG**		**IFN-γ**	

**Group**	Pig #*	**d11-48**	**d67-75**	**d83/84**	**d6-48**	**d67-75**	**d6-48**	**d67-75**	**d11-48**	**d60-82**

RE	37	+	**++**	+	**++**	**++**	+	+	+	**++**
RE	38	+	+		**++**	+	**++**	**++**	+	**++**
RE	52	+	**++**	**++**	+	**++**	+	**++**	+	**++**
RE	55	+	**++**	**++**	+	**++**	+	**++**	+	**++**
RE	57		+		+	+	**++**	**++**	+	**++**
RE	26	+	+	+	+	+	+	**++**	+	**++**
RE	39		+	+	+	+	+	+	+	**++**
RE	53	+	+		+		+	**++**	+	**++**
RE	61	+	**++**		+	+	**++**	+	+	**++**
RE	64		+		+	+		+	+	**++**

CC	36		**++**			+		**++**		+
CC	44			+		**++**		**++**		+
CC	51		**++**			**++**		**++**		**++**
CC	58		+	**++**		+		**++**	+	+
CC	59		+	+		**++**	+	**++**		+
CC	66	+	**++**	**++**		**++**		**++**		**++**
CC	70		+	+		**++**		**++**		+

The level of response for *L. intracellularis*-specific IgA in faeces and ileum content, IgA and IgG in serum, and antigen-specific IFN-γ-production in whole blood cultures were scored for individual pigs from the RE group and the CC group. Summed scores for the primary and secondary infection periods are categorised as: no response (BLANK), low-medium response (+), high response (++). * All pigs in the RE group were protected against re-infection as defined by: absence of bacterial shedding and serum acute phase protein response as well as low or no IHC score at termination of the experiment (see Result section and [6]).

## Results

### *L. intracellularis*-specific serum IgG and IgA

**Figure 1:**
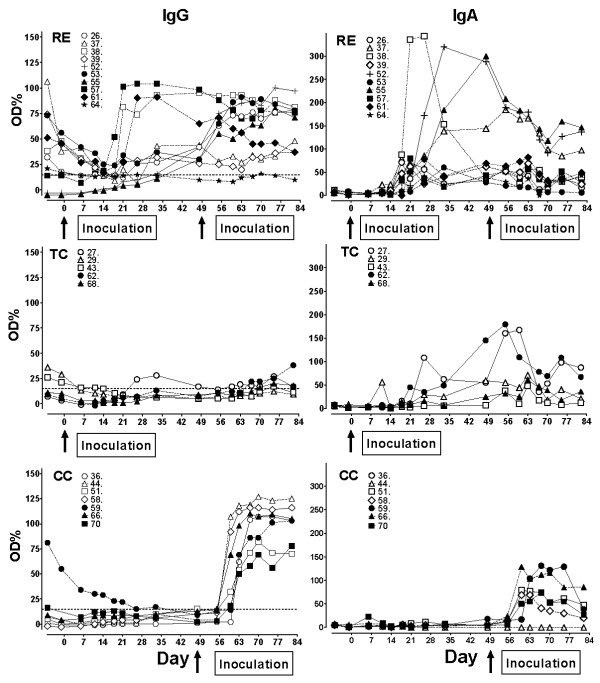
***L. intracellularis*-specific IgA and IgG in serum**. Sera were collected at the time points indicated from *L. intracellularis*-inoculated pigs and IgA and IgG was measured by ELISA (diluted 1:12.5 and 1:50, respectively). Values from individual pigs in group RE, TC, and CC are expressed as OD% of positive control sera of individual animals. Arrows indicate inoculation with *L. intracellularis *at day 0 and at day 49.

Percentages of IgG positive pigs before and after challenge inoculation for the three groups has been published previously [6]. Here we present the levels of *L. intracellularis*-specific IgG and IgA in the serum of individual pigs (Figure 1) in order to detail the primary and secondary immune responses to infection. The IgG response profiles in the RE group were variable with some pigs showing a gradually increasing response after primary infection, which further increased after challenge. Other RE pigs mounted a strong primary IgG response that stayed high throughout the experiment and still other pigs were low-responders barely mounting a response above the ELISA cut-point and without a boosting effect of the challenge. In the TC group the IgG responses to the primary inoculation were low, if the pigs were at all positive, while all naive CC pigs responded to the challenge (day 49) with a marked IgG response in serum. Some pigs in all groups had maternal antibodies which gradually decreased and did not appear to influence the generation of new IgG antibodies.

Levels of *L. intracellularis*-specific IgA in serum increased after the primary *L. intracellularis *infection at day 0 in RE and TC pigs (Figure 1). Except for a few pigs (*n *= 3/10), serum IgA in RE pigs levelled out slowly after the peak at 3-4 weeks post inoculation (pi), and was almost at background level at the end of the experiment irrespective of the challenge inoculation at day 49 pi. The pigs in the TC group had a serum IgA profile similar to the RE group, indicating that there was no boosting effect of re-infection. Pigs in the CC group responded to the challenge inoculation at day 49 with a strong IgA response in serum compared to the primary response seen in RE and TC pigs.

### Total- and *L. intracellularis*-specific IgA in faeces

*L. intracellularis*-specific IgA was measured in faeces extracts and standardised to the amount of total IgA for each individual sample. Total faecal IgA was very low (< 200 ng/mg faeces extracts) in all groups until day 33 when the pigs were approximately 10 weeks old, indicating that the faecal IgA response is immature in young pigs. After that time point total faecal IgA increased in all groups (data not shown).

**Figure 2:**
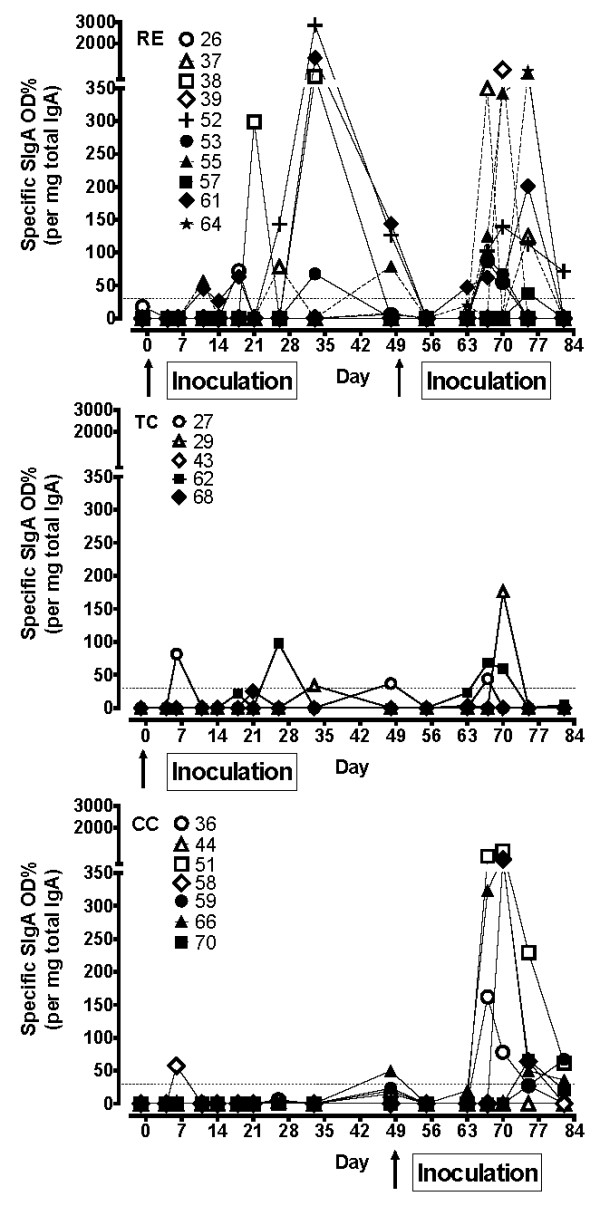
***L. intracellularis*-specific IgA in faeces**. Faeces samples were collected at the time points indicated and *L. intracellularis*-specific IgA was measured by ELISA. Values for specific IgA are expressed as OD% of positive control serum/μg total IgA/mg dry faeces of the individual pig from group RE, TC and CC. Arrows indicate inoculation with *L. intracellularis *at day 0 and at day 49.

The level of *L. intracellularis*-specific faecal IgA relative to total faecal IgA is shown in Figure 2. Among RE pigs 7/10 responded to the primary inoculation with measurable faecal IgA. In response to the challenge inoculation 16/17 RE and CC pigs had detectable faecal IgA at days 67, 70 or 75, but not at day 82. There was no difference in the levels of faecal IgA responses between the protected RE group and the susceptible CC group. In the TC group only 3 out of 5 pigs had positive but low responses above background.

IgA was also measured in ileum content collected at day 83 or 84. Five samples were positive out of 10 in the RE group and 5 out of 7 in the CC group (Table 1). There were no differences between the groups.

### *L. intracellularis*-specific cell-mediated immune responses

**Figure 3:**
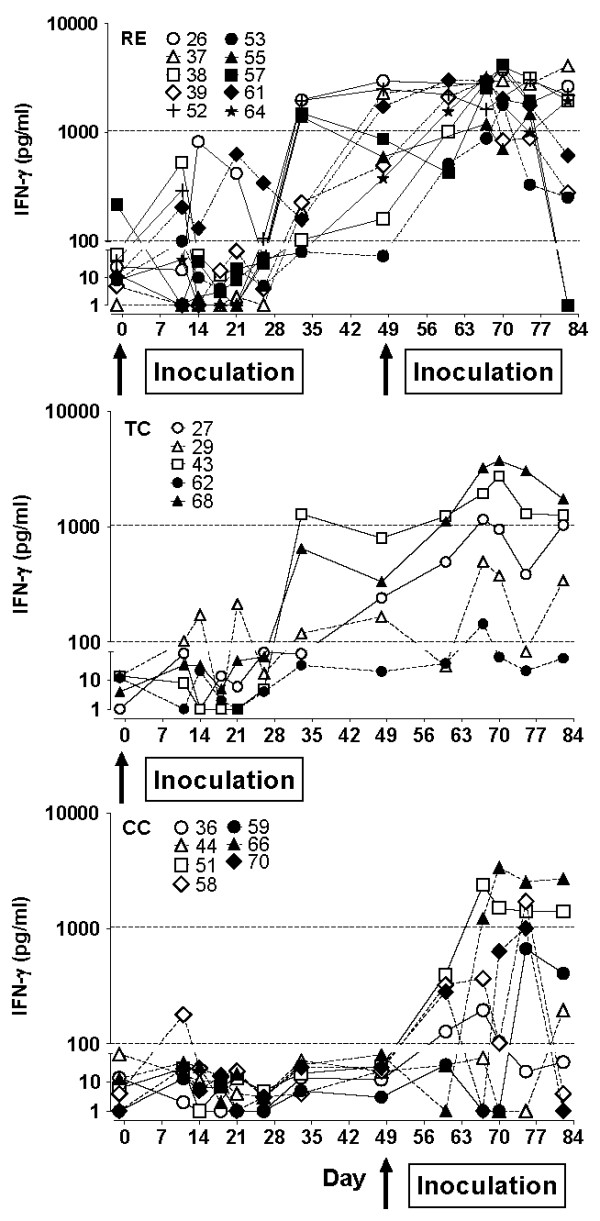
***L. intracellularis*-specific IFN-γ production**. *L. intracellularis*-specific IFN-γ production by whole-blood cultures co-stimulated with IL-18. Graphs present IFN-γ responses from individual pigs at the time points indicated in group RE, TC, and CC. Arrows indicate inoculation with *L. intracellularis *at day 0 and at day 49.

The *L. intracellularis*-specific IFN-γ responses in whole blood after primary *L. intracellularis *inoculation and challenge are presented in Figure 3. Following primary infection of 5-6 week old piglets (RE and TC groups), the initial IFN-γ responses were moderate with around 50% of the pigs responding with Ag-specific IFN-γ above background level (100 pg/mL), and with several pigs showing a sustained high Ag-specific IFN-γ response even at day 48 pi. From the TC group it appears that Ag-specific IFN-γ response in some pigs peaks 9-10 weeks after primary infection, and may develop even in pigs that did not show IFN-γ response immediately after the inoculation. After challenge at 12-13 weeks of age, an increase in the IFN-γ responses of RE pigs compared to pre-challenge levels (day 33 and 48) were observed at day 60 through 75 with statistically significant difference at days 67 and 70 (*P *< 0.05, paired *t*-test) and near statistical significant difference at days 60 and 75 (*P *= 0.065 and 0.090, respectively). While some TC pigs had peak levels of IFN-γ at the same time these were not statistically significantly different from day 33 or 48. Following primary infection of 12-13 week old pigs (CC group), Ag-specific IFN-γ responses appeared earlier and with higher levels compared to the responses in the younger piglets. The level of recall memory IFN-γ responses in RE pigs were, however, statistically significantly higher than the primary response of CC pigs until 26 days post challenge (day 75) (*P *< 0.01 days 60 and 67, and *P *< 0.05 day 70 irrespective of unpaired *t*-test or Mann-Whitney).

### Phenotypes of IFN-γ producing lymphocytes

**Figure 4:**
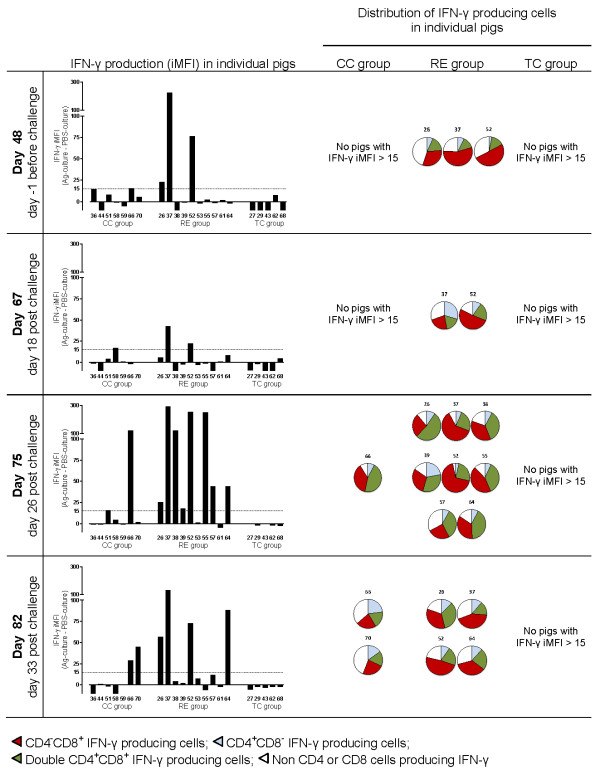
**Phenotypes of *L. intracellularis*-specific IFN-γ producing cells**. Flow cytometry characterization of *L. intracellularis *specific IFN-γ producing cells in individual pigs from the RE group, TC group and CC group, measured before challenge (day 48) and after challenge (day 67, 75, and 82). Bars show levels of IFN-γ responses in lymphocytes (iMFI (Ag-culture) subtracted iMFI (PBS-culture)), and for iMFI > 15, the distribution of IFN-γ producing cells in Ag-cultures are shown.

Figure 4 shows the *L. intracellularis*-specific IFN-γ response in lymphocytes (iMFI) measured by flow cytometry together with the distribution of IFN-γ producing cells (CD4 and CD8 profile). At 48 days after primary inoculation *L. intracellularis*-specific IFN-γ response (iMFI in gated lymphocytes) could be detected in three pigs (#26, #37, #52), and not in the non-inoculated control pigs. After challenge inoculation, increased IFN-γ iMFI was detected in several pigs in the RE group especially at day 75 (8 of 10 pigs) and 82 (4 of 10 pigs). In contrast to the IFN-γ whole blood assay, flow cytometry could not detect *L. intracellularis*-specific IFN-γ (iMFI responses above 15) in the five TC pigs at these time points. Increased IFN-γ iMFI was measured in 2 of 7 CC pigs (#66, #70) at day 75 or 82, corresponding to day 26 and 33 after their primary *L. intracellularis *challenge.

Identification of phenotypes of the cells producing IFN-γ after culture with *L. intracellularis *antigen showed in most cases a high fraction of CD8^+^(CD4^-^) cells and/or CD4^+^CD8^+ ^double positive cells whereas only a low fraction of cells were identified as CD4^+^(CD8^-^). In addition, some cells which were neither CD4 nor CD8 positive contributed to IFN-γ production (Figure 4). The phenotypes of IFN-γ producing cells after Ag-specific stimulation were not a reflection of the general distribution of phenotypes in un-stimulated cells (PBS-cultures). As an example (mean distribution +/- SEM in RE group), on day 75 the % of IFN-γ^+^CD4^+^CD8^+ ^cells in Ag-cultures were significantly different from % of CD4^+^CD8^+ ^in PBS-cultures (35.1% +/- 3.2% vs. 5.5% +/- 0.8%, *P *= 0.0001, paired *t*-test) and for IFN-γ^+^CD4^-^CD8^+ ^cells (40.7% +/- 5.7% vs. 23.2% +/- 1.8%, *P *< 0.01), respectively. Accordingly, % of CD4^+^CD8^- ^cells were significantly reduced (9.3% +/- 1.9% vs. 21.3% +/- 1.4%, *P *< 0.01). High CD25 expression was mainly identified in the population of CD4^+^CD8^+ ^double positive cells, and especially in those cells producing IFN-γ (data not shown).

**Figure 5:**
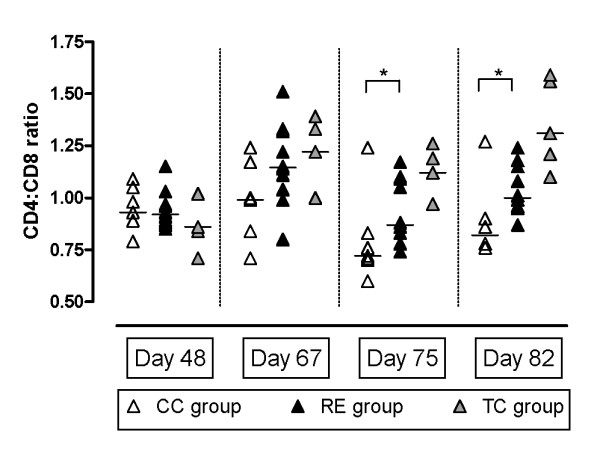
**CD4:CD8 ratios in cell cultures**. CD4:CD8 ratios (mean of Ag-culture and PBS-culture) of gated lymphocytes in PBMC cell cultures before challenge (day 48) and after challenge (day 67, 75 and 82). Median ratios (line) and significant differences (*) are shown for CC group and RE group.

The CD4:CD8 ratios within gated lymphocytes in the PBMC cultures are shown in Figure 5. In response to the inoculation at day 49 a significantly reduced CD4:CD8 ratio was found in the susceptible CC group compared to the protected RE group at day 75 and 82 (*p *< 0.05 Mann Whitney and unpaired *t*-test). No significant differences were observed between the CC and RE groups at days 48 and 67.

### Characterisation of intestinal cell subsets

**Figure 6:**
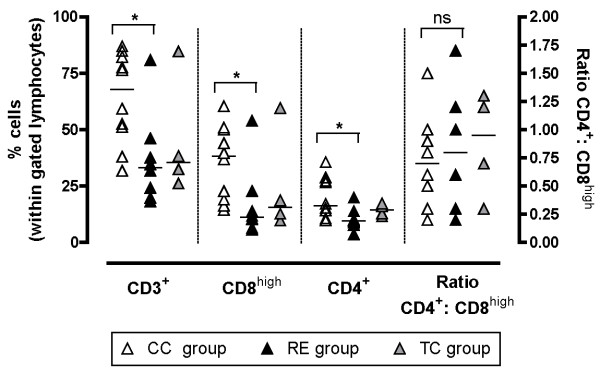
**Distribution of T cell subsets isolated from lamina propria**. Distribution of lamina propria T cell subsets 3 weeks pi analysed by flow cytometry within a tight gate of lymphocytes defined from forward and side scatter profile. Bars indicate median value. * indicates *P *< 0.05 (Mann-Whitney).

The distributions of lymphocyte phenotypes in the jejunum of pigs in the preceding Experiment I was analyzed at autopsy around 3 weeks after challenge (Figure 6). It was not possible to perform Ag-specific cultures of intestinal cells without bacterial contamination and thus Ag-specific IFN-gamma producing cells could not be identified. CD8^+ ^cells were analysed for high expression (CD8^high^), since this population is expected mainly to comprise MHC-class I restricted CTL [21]. Analysis of phenotype distributions in LPC suspensions revealed significant increased percentages of CD3^+^, CD8^high ^and CD4^+ ^cells in the CC group compared to the RE group, but without significant difference in CD4:CD8^high ^ratios. Low levels of CD25^+ ^cells were identified in the cell suspensions from all pigs (data not shown). Analysis of IEC did not reveal significant differences in percentages of CD3^+^, CD8^high ^and CD4^+ ^cells in IEC suspensions between the CC and RE groups (data not shown). However, in three CC pigs with macroscopic signs of proliferative enteropathy at autopsy, increased levels of CD172a positive cells were observed mainly in IEC suspensions (data not shown), indicating increased presence of macrophages and/or granulocytes.

### Scoring of immune response parameters

In Table 1 all measured immune response parameters were scored relative to the median response levels and summarized for the primary (CC pigs) and secondary protective (RE pigs) immune responses to *L. intracellularis *inoculation in age matched pigs. All of the protected RE pigs had high scores on CMI (IFN-γ) between day 67 and 75. In contrast only 2 of 7 susceptible CC pigs had high IFN-γ score and the rest had low-medium score in that time period. The higher numbers of Ag-specific IFN-γ high-responses in the RE pigs compared to CC pigs at this time was significantly different (Fisher exact test, *P *= 0.0034). With regards to the other host response parameters, most pigs had high systemic and local humoral immune responses between days 67-75, although with a tendency to fewer serum IgA-high responders in the RE group (3 of 10 pigs (30%)) compared to the CC group (5 of 7 pigs (83%). This was not reflected in the number of SIgA responders, however, which were at the same level at day 67-75 in the RE group (4 of 10 high-responders (40%)) and the CC group (3 of 7 high-responders (43%)).

## Discussion

The immune response to a subclinical primary *L. intracellularis *infection is highly variable between pigs infected at different ages. The IFN-γ response to infection in 5-6 week old piglets was low or absent in the present study as with observations from other studies [14]. However, a delayed *L. intracellularis*-specific IFN-γ response was observed at days 33 and/or 48 pi continuing up to 12 weeks pi even in TC pigs. In contrast, the older CC pigs inoculated at 12-13 weeks of age mounted significant *L. intracellularis*-specific IFN-γ responses already from day 14 pi. The RE pigs were challenged with *L. intracellularis *in parallel with the CC pigs, and exhibited an immediate boost in IFN-γ response, which was significantly higher than CC pigs and peaked 2-3 weeks pi. This boosting of CMI after challenge inoculation was corroborated by increased iMFI measurements in RE pigs compared to both CC and TC pigs and suggests a role for CMI in the observed protection against re-infection with *L. intracellularis*.

Since *L. intracellularis *reside and multiply in the cytosol of infected cells [24], peptides of bacterial origin will be presented on the surface of infected cells in MHC class I complexes making these cells potential targets of CD8^+ ^cytotoxic T cells. In immune pigs we observed high numbers of *L. intracellularis*-specific IFN-γ producing CD8^+ ^cells as contributors of the observed IFN-γ response in blood. In susceptible CC pigs a relative expansion of the CD8^+ ^cell population (reduced ratio of non-specific CD4:CD8 T cells) was observed 3-4 weeks after their primary encounter with the infection. CC pigs in a preceding experiment also experienced a relative increase in both CD4^+ ^and CD8^+ ^cells in intestinal samples compared to the protected RE pigs at three weeks post challenge inoculation. Together this may indicate that CD8^+ ^lymphocytes are important local effector cells in protective immunity against *L. intracellularis *at the intestinal level. No specific surface staining to separate CTL from NK cells was performed, and the IFN-γ producing CD8α^+ ^(CD4^-^) lymphocytes may thus include cells of the NK-family. Both CTL and NK cells can respond to IL-18 potentiation and produce IFN-γ, but only CTL have the Ag-specificity observed in both FACS and IFN-γ assays in the present study. Thus CTL is the most likely candidate for the *L. intracellularis *responsive IFN-γ^+^CD8^+ ^phenotype. However, since no studies on cytotoxic effector functions were performed, it remains speculation as to whether this *L. intracellularis*-specific IFN-γ^+^CD8^+ ^population in immune pigs have effector functions against infected cells. Although CD8^+ ^CTL appear to be responsible for some of the IFN-γ production observed in the whole blood assay, the CD4^+^CD8^+ ^double positive cells produce higher levels of IFN-γ in response to *L. intracellularis*-specific stimulation [18] in line with their suggested role as memory helper T cells [21,25,26]. This is the first report that demonstrates development of *L. intracellularis*-specific lymphocytes consistent with effector and memory T cell phenotypes which may be involved in protective immune mechanisms against this bacterium.

Secretory IgA is the primary immunoglobulin isotype at mucosal surfaces (reviewed by Woof and Kerr [27]), and locally produced *L. intracellularis*-specific IgA induced by a primary infection may be important in protection against re-infection. Previous studies have associated mucosal *L. intracellularis *infection with accumulation of IgA in affected areas of the intestine [13] and *L. intracellularis*-specific IgA has been measured in samples from ileum lavage at day 22 post *L. intracellularis *infection of pigs at 5 weeks of age [14]. In the present study it was not possible to identify a significant boost in *L. intracellularis*-specific serum IgA in RE pigs following the challenge inoculation. The levels of serum IgA in these pigs decreased in a pattern that was comparable to the non-challenged but primary infected pigs (TC) while the majority of pigs in the CC group responded to the infection with a marked serum IgA response. Serum IgA is metabolized some 5 times faster than serum IgG [27] and accounts for the relatively short-lived IgA response compared to IgG. While only the CC group of pigs responded to the challenge inoculation with serum IgA, both CC and RE pigs responded to this inoculation with strong faecal IgA responses from days 63 to 84. The intestinal IgA system lacks classical immune memory characteristics [28] and the induction of IgA in faeces of the protected RE pigs in response to the challenge inoculation may be evidence of renewed activation of the local intestinal immune response. It is not possible to measure whether faecal IgA represents locally produced secretory IgA or transudations from serum, but measurements of faecal IgA were corroborated by measurements of local IgA in the ileum at autopsy (Table 1).

Although we correlated *L. intracellularis*-specific faecal IgA to total faecal IgA and corrected for variation in faecal water content using freeze dried faeces, there were still sources of error in these measurements resulting from large variations in secretion of IgA and probably the inhomogeneous faecal composition [29]. With a dry weight content around 30% for all groups of animals (data not shown), the levels were comparable to published total IgA levels between 10-100 ng/mg wet faeces in pigs aged 4-7 weeks [30].

As in other *L. intracellularis *experimental infection studies [17,31], we found IgG seroconversion 2-3 weeks after primary infection at either 5-6 weeks or 12 weeks of age and with higher levels of IgG responses in pigs infected at 12 weeks compared to the younger pigs. However, at the time of challenge, 7 weeks after the primary infection, pig #64 did not have *L. intracellularis*-specific IgG levels in serum samples above the ELISA cut point, but was equally well protected compared to other RE pigs. Furthermore, not all of the RE pigs experienced a boost in serum IgG upon secondary infection, although all RE pigs were protected against the infection. Thus, the presence or absence of serum IgG antibodies, specific for the DOC extracted *L. intracellularis *antigens employed in the ELISA, before or after the secondary infection did not correlate with the observed protection. Likewise, the maternal antibodies present in nearly half of the pigs were not protective against the primary *L. intracellularis *inoculation at 5 weeks of age. Thus, the importance of serum IgG in the observed protection against *L. intracellularis *is questionable and in line with vaccination studies where protective immunity against *L. intracellularis *has been shown to develop without a robust IgG antibody response [32].

Infection with *L. intracellularis *in European pig herds is mainly a problem in younger age groups [7,8]. From our observations it is clear that the older CC pigs develop a faster and more comprehensive immune response, involving CMI as well as local and systemic humoral responses, to a primary infection compared to the young RE or TC piglets. This suggests that reduced susceptibility to *L. intracellularis *with age may be related to maturation of the immune system and particularly to the generation of CMI responses. It is also noteworthy that in the first part of the experiment very low total faecal IgA was measured in piglets, which was also reflected in very low levels of *L. intracellularis*-specific faecal IgA. The low IgA content in faeces may be further contributing to making piglets especially vulnerable to infections.

In conclusion, it is possible to speculate on the relative role of the different immune responses in the observed protection: Serum IgG, whether maternally derived or produced by the pig, did not appear to correlate with protection and there was no difference in level of protection between pigs with or without increased levels of serum IgG in response to the challenge inoculation. Local intestinal IgA antibodies may be involved in the protection against *L. intracellularis*, but we observed identical faecal IgA response profiles between CC and RE pigs emerging two to four weeks post challenge inoculation in line with the reported lack of immune memory in the intestinal IgA system [28]. Together with the absence of an acute phase protein response to the challenge inoculation in protected pigs, it appears logical to conclude that protection was mediated before intestinal IgA was induced. Increased responses in the IFN-γ assay were, however, observed at the first sampling post challenge and RE pigs responded significantly stronger to the challenge inoculation than CC pigs indicating a CMI memory recall response. Flow cytometry analyses further identified antigen-specific CD8^+ ^and CD4^+^CD8^+ ^double positive lymphocytes as contributors of the released IFN-γ, which suggests these cell types may be central components in immune mediated protection against *L. intracellularis *infection.

## List of abbreviations

RE: re-infection group; TC: treatment control group; CC: challenge control group; CMI: cell mediated immunity; DTH: delayed type hypersensitivity; PBMC: peripheral blood mononuclear cells; CTL: Cytotoxic T lymphocyte; DOC: deoxycholate; SEB: Staphylococcus enterotoxin B; PBS: phosphate-buffered saline; iMFI: integrated median fluorescence intensity

## Competing interests

The authors declare that they have no competing interests.

## Authors' contributions

HC developed and carried out the serological tests and drafted and participated in writing the manuscript. UR designed and carried out the experimental animal study and the cellular assays and participated in writing the manuscript. TKJ participated in study design, autopsies and collection of samples from the animal experiments. GJ participated in study design, coordination of the study, data analysis and in writing the manuscript. All authors read and approved the final manuscript.
